# Editorial: Acute Kidney Injury: It's Not Just Acute, and It's Not Just the Kidneys

**DOI:** 10.3389/fped.2021.792210

**Published:** 2021-11-15

**Authors:** Danielle E. Soranno, Akash Deep, Katja M. Gist, Michael Zappitelli

**Affiliations:** ^1^Section of Pediatric Nephrology, Departments of Pediatrics, Bioengineering and Medicine, University of Colorado, Aurora, CO, United States; ^2^Department of Child Health, NHS Foundation Trust, King's College Hospital, London, United Kingdom; ^3^Division of Pediatric Cardiology, Department of Pediatrics, University of Cincinnati, Cincinnati, OH, United States; ^4^Division of Nephrology, Department of Pediatrics, Hospital for Sick Children, Toronto, ON, Canada

**Keywords:** acute kidney injury, pediatric nephrology, Continuous Renal Replacement Therapies (CRRT), diabetes mellitus, Artificial Intelligence-AI, metabolic bone disease, critical care nephrology

Acute kidney injury (AKI) occurs in ~20, 30, and 40–60% of pediatric patients in pediatric, neonatal and cardiac intensive care units, respectively (1, 2). Twenty years ago, AKI in hospitalized children was considered an unfortunate consequence of illness, but ultimately self-limited without direct impacts on patient outcomes. Extensive research in the last 15 years revealed that AKI in hospitalized children is associated with greater hospital resource utilization, morbidity and hospital mortality (1, 3). Shockingly, many episodes of pediatric hospital-acquired AKI continue to go unrecognized (4). More recently, emerging evidence suggests that children surviving hospitalization with an AKI episode may be at substantially higher risk than children without AKI, for developing long-term chronic kidney disease (CKD) and hypertension and long-term mortality (5). This literature has contributed to a paradigm shift that AKI increases risk for poor long-term outcomes, warranting efforts to improve detection, management and secondary prevention of permanent sequelae.

The goals of this collection of 9 manuscripts are to provide an update on best practices for detection, management, and follow-up of pediatric AKI, and perspectives on the deleterious short- and long-term systemic sequelae of AKI in the context of evolving clinical, epidemiologic and fundamental AKI research.

In Impact of Acute Kidney Injury on Critically Ill Children and Neonates, authors Leghrouz and Kaddourah provide an overview of the recent shifts in AKI definition, now centering on the KDIGO defined criteria using either a rise in serum creatinine, and/or a reduction in urine output. The authors highlight the importance of measuring urine output in order to recognize oliguric AKI for improved diagnostic precision. The review also provides a summary of the impact of AKI following hematopoietic stem cell transplant and emerging data on children with multisystem inflammatory syndrome as a result of the SARS-CoV-2 pandemic.

In Acute Kidney Injury in Critically Ill Children Is not all Acute: Lessons Over the Last 5 Years, Hessey et al. reviewed the last 5 years of peer-reviewed literature exploring the long-term outcomes of pediatric AKI. The summary provides an overview of the advancements and remaining challenges in investigating long-term outcomes after critical illness in hospitalized children. It also highlights the importance of implementing standardized follow-up of kidney health and outcomes after an episode of severe AKI. This aligns with recent recommendations in the neonatal and pediatric response to the Acute Disease Quality Initiative (ADQI) 22 guidance document (6).

In A Precision Medicine Approach to Biomarker Utilization in Pediatric Sepsis-Associated Acute Kidney Injury, authors Odum et al. reframe sepsis-associated AKI as a heterogeneous disease process. They provide a framework approach to utilizing biomarkers for prognostic enrichment and precision medicine to decluster the sepsis phenotype into distinctive pathophysiologies for targeted therapeutic clinical trials. This manuscript highlights the need to improve our understanding of AKI phentoypes for which future therapeutic targets might be considered.

In Continuous Renal Replacement Therapy in Critically Ill Children in the Pediatric Intensive Care Unit: A Retrospective Analysis of Real-Life Prescriptions, Complications, and Outcomes, Buccione et al. present new data from a 6-year single center retrospective study evaluating outcomes after CRRT therapy in pediatrics. Their data reports that fluid overload was the predominant indication for CRRT initiation (>60% of cases) and identifies catheter size <8 French and lack of citrate regional anticoagulation as predictors of early discontinuation of treatment. With a mean follow-up time of 3.5 years, only 42% of the patients who survived did not have any long-term kidney sequelae, including CKD or proteinuria.

In Two to Tango: Kidney-Lung Interaction in Acute Kidney Injury and Acute Respiratory Distress Syndrome, Alge et al. provide a thorough review of the basic science and clinical data surrounding the development of AKI-mediated lung injury and the role that lung injury plays in affecting kidney function. The review highlights the complexity of identifying the bidirectional effects of hemodynamic alterations in critical illness, resulting from acute lung disease as well as from AKI.

In A review on the application and limitations of administrative health care data for the study of acute kidney injury epidemiology and outcomes in children, Ulrich et al. summarize the opportunities and limitations of leveraging “big data” in administrative health repositories to investigate pediatric AKI outcomes. While big data research offers unique and unprecendented opportunities to evaluate pediatric AKI with large sample sizes, there is a need to validate these data for defining pediatric kidney outcomes and limiting associated biases.

In For Whom the Bell Tolls: Acute Kidney Injury and Electronic Alerts for the Pediatric Nephrologist, authors Nguyen and Menon review best practices for AKI alerts, and how they can be implemented to hasten the diagnosis and optimize management in pediatric AKI. These AKI alerts and tools tie in with broader efforts within the nephrology community to utilize artificial intelligence and machine learning to improve outcomes in patients with AKI. The review provides examples of early applications of alert tools in pediatric AKI, including a nephrotoxic-mediated AKI stewardship program, which has been shown to reduce the rate, severity and duration of nephrotoxic AKI (7, 8).

In Acute kidney injury in pediatric diabetic kidney disease, Piani et al. describe the pathophyisiology of AKI and its relation to diabetic kidney disease in children with types I and II diabetes mellitus. Children with diabetes are at an increased risk for developing AKI, and after an episode of AKI, they are at higher risk of developing diabetic kidney disease and CKD. The review also summarizes the increased risk of severe COVID-19 disease in patients with diabetes mellitus.

In Acute Kidney Injury and Pediatric Bone Health, authors Hegde et al. review the current literature on fracture risk after AKI, the mechanisms of dysregulation in bone metabolism in AKI, and suggest areas for future research, particularly with regard to skeletal growth in pediatric patients after AKI.

As the overall survival of critically ill pediatric patients improves, there are increasing survivors of childhood AKI. With these perspectives in mind, future research is required to explore the long-term impact of pediatric AKI on population health, health care costs, and how pediatric providers within varying healthcare contexts, can potentially improve care delivery with a goal to optimize their patients' outcomes later in life.

## Author Contributions

DS, AD, KG, and MZ contributed to the editorial writing and revision process. All authors contributed to the article and approved the submitted version.

## Conflict of Interest

The authors declare that the research was conducted in the absence of any commercial or financial relationships that could be construed as a potential conflict of interest.

## Publisher's Note

All claims expressed in this article are solely those of the authors and do not necessarily represent those of their affiliated organizations, or those of the publisher, the editors and the reviewers. Any product that may be evaluated in this article, or claim that may be made by its manufacturer, is not guaranteed or endorsed by the publisher.
